# Antimicrobial Activity and Cell Selectivity of Synthetic and Biosynthetic Cationic Polymers

**DOI:** 10.1128/AAC.00469-17

**Published:** 2017-09-22

**Authors:** Mayandi Venkatesh, Veluchamy Amutha Barathi, Eunice Tze Leng Goh, Raditya Anggara, Mobashar Hussain Urf Turabe Fazil, Alice Jie Ying Ng, Sriram Harini, Thet Tun Aung, Stephen John Fox, Shouping Liu, Liang Yang, Timothy Mark Sebastian Barkham, Xian Jun Loh, Navin Kumar Verma, Roger W. Beuerman, Rajamani Lakshminarayanan

**Affiliations:** a Anti-Infectives Research Group, Singapore Eye Research Institute, The Academia, Singapore; b Ophthalmology and Visual Sciences Academic Clinical Program, Duke-NUS Graduate Medical School, Singapore; c Lee Kong Chian School of Medicine, Nanyang Technological University, Experimental Medicine Building, Singapore; d Bioinformatics Institute, Agency for Science, Technology and Research (A*STAR), Singapore; e Singapore Centre for Environmental Life Sciences, Nanyang Technological University, Singapore; f School of Biological Sciences, Nanyang Technological University, Singapore; g Department of Laboratory Medicine, Tan Tock Seng Hospital, Singapore; h Institute of Materials Research and Engineering, A*STAR, Singapore

**Keywords:** antimicrobial activity, cationic polymers, cell selectivity, membrane selectivity, rapid bactericidal activity, superior biocompatibility index, topical bacterial infections

## Abstract

The mammalian and microbial cell selectivity of synthetic and biosynthetic cationic polymers has been investigated. Among the polymers with peptide backbones, polymers containing amino side chains display greater antimicrobial activity than those with guanidine side chains, whereas ethylenimines display superior activity over allylamines. The biosynthetic polymer ε-polylysine (εPL) is noncytotoxic to primary human dermal fibroblasts at concentrations of up to 2,000 μg/ml, suggesting that the presence of an isopeptide backbone has greater cell selectivity than the presence of α-peptide backbones. Both εPL and linear polyethylenimine (LPEI) exhibit bactericidal properties by depolarizing the cytoplasmic membrane and disrupt preformed biofilms. εPL displays broad-spectrum antimicrobial properties against antibiotic-resistant Gram-negative and Gram-positive strains and fungi. εPL elicits rapid bactericidal activity against both Gram-negative and Gram-positive bacteria, and its biocompatibility index is superior to those of cationic antiseptic agents and LPEI. εPL does not interfere with the wound closure of injured rabbit corneas. In a rabbit model of bacterial keratitis, the topical application of εPL (0.3%, wt/vol) decreases the bacterial burden and severity of infections caused by Pseudomonas aeruginosa and Staphylococcus aureus strains. *In vivo* imaging studies confirm that εPL-treated corneas appeared transparent and nonedematous compared to untreated infected corneas. Taken together, our results highlight the potential of εPL in resolving topical microbial infections.

## INTRODUCTION

In the era of increasing antibiotic resistance, antiseptics are valuable alternatives for the management of topical wounds and infections. Antiseptics are antimicrobial agents that can rapidly inhibit or destroy the growth of microorganisms and are an important component of infection control and prevention in hospitals and health care settings (1–3). Their broad-spectrum antimicrobial activity, rapid inhibitory or microbicidal activity, and nonspecific mode of action potentially slow the evolution of antimicrobial resistance. Antiseptic agents vary widely in terms of chemical structures, molecular masses, antimicrobial properties, and modes of action (3, 4). Ideal agents prevent microbial colonization without inhibiting host cell proliferation or tissue regeneration. However, in general, although available agents display rapid microbicidal properties, many remain cytotoxic to mammalian cells (5–7). In chronic wounds, where the polymicrobial flora and the presence of biofilms and high bacteria loads induce a host immune response and delay the healing process, the use of available antiseptics can add insult to injury.

The recent ban on wash products containing topical antiseptics by the U.S. FDA, due to their hazards to human health, has highlighted the need for alternative antimicrobial agents (8). A study on commercially available antiseptic agents showed that these agents have a significant cytotoxic effect on fibroblasts at concentrations required to decrease the bacterial bioburden (9). That study furthermore demonstrated that polyhexamethylene biguanides (PHMBs) and octenidine showed the highest biocompatibility index compared with antiseptics containing silver or povidone iodine. The potent antiseptic properties of PHMB and octenidine are attributed to their cationic nature and strong affinity for negatively charged components of the cell wall and cytoplasmic membranes of bacteria (10, 11). However, their utility as topical antibacterials is limited due to their irritant and allergic properties at elevated concentrations (12, 13). There remains an unmet need for antiseptic agents that have a higher biocompatibility index, especially for the management of chronic wounds.

**FIG 1:**
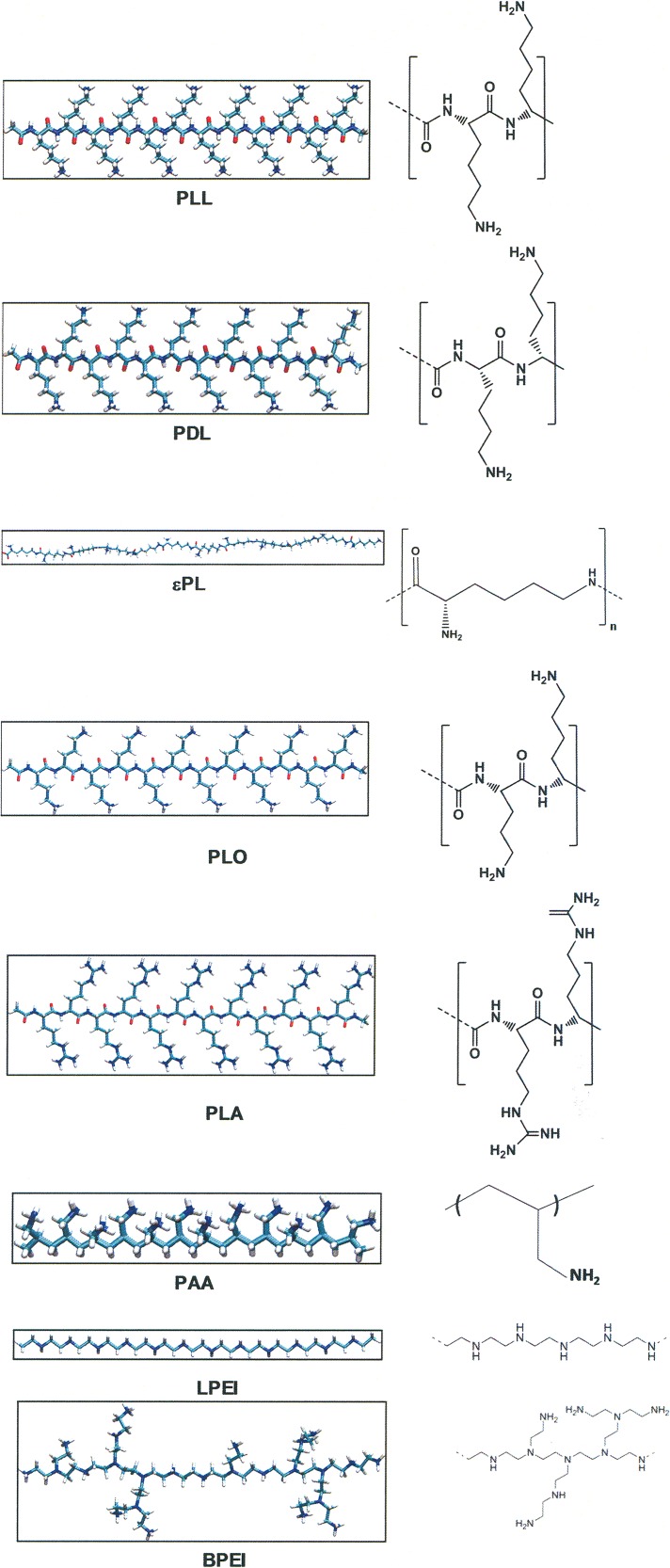
Chemical structures of cationic polymers used in this work. Shown are stick models and chemical structures of the polymers used in this work. The models were prepared with Discovery studio using VMD and are depicted in stick representation and colored (white, hydrogens; cyan, carbons; blue, nitrogen; red, oxygen). Abbreviations: εPL, epsilon polylysine; PLL, α-poly-l-lysine; PLO, α-poly-l-ornithine; PDL, α-poly-d-lysine; PLA, α-poly-l-arginine; PAA, poly(allylamine); LPEI, linear polyethylenimine; BPEI, branched polyethylenimine.

Cationic polymers are an attractive prospect, with their rapid microbicidal properties, increased bioavailability, and chemical diversity (14–18). They target the negatively charged cytoplasmic membranes of microbes, like cationic biocides and host defense peptides, but higher concentrations are required to elicit a cytotoxic effect on mammalian cells (19–21). The purpose of this article is to investigate the antimicrobial and cytotoxic properties of cationic polymers with peptide/isopeptide and polyethylene/polyethylenimine (PEI) backbones (Fig. 1). Furthermore, we investigated the broad-spectrum antimicrobial properties, time-kill kinetics, antibiofilm properties, *in vivo* efficacy, and toxicity of a polymer that showed good antimicrobial activity but remained noncytotoxic to mammalian cells.

## RESULTS

### Antimicrobial properties and cytocompatibility of cationic polymers.

Table 1 shows the MIC values of cationic polymers against 5 different strains of Staphylococcus aureus, Pseudomonas aeruginosa, and Candida albicans as well as the biocompatibility for primary human dermal fibroblasts (hDFs). We compared the antimicrobial properties and cytotoxicities of the polymers with those of the cationic antiseptics benzalkonium chloride (BAK) and chlorhexidine (CHX). For polymers with a peptide backbone (except α-poly-l-arginine [PLA]), the MIC values against Gram-positive/Gram-negative pathogens ranged from 16 to 1,024 μg/ml. Among the polymers with a polyethylene or ethylenimine backbone, linear polyethylenimine (LPEI) displayed superior antimicrobial activity against bacterial pathogens over poly(allylamine) (PAA). For polymers containing peptide or isopeptide bonds, however, higher MIC values against C. albicans than against bacteria were observed, suggesting that the presence of a β-d-glucan cell wall structure or proteases in yeasts confers greater protection against these agents. However, no such shift in the MIC values was observed for LPEI/branched polyethylenimine (BPEI) polymers. The cationic antiseptic agents (BAK and CHX) had lower MIC values against S. aureus and C. albicans than against P. aeruginosa, and the antimicrobial activity was superior to those of cationic polymers in terms of mass concentration (Table 1).

**TABLE 1:** MICs of cationic polymers against various bacterial and yeast strains and their cytotoxicity to primary human dermal fibroblasts

Strain	MIC (μg/ml)^*a*^									
	PLL	PDL	εPL	PLO	PLA	PAA	LPEI	BPEI	BAK	CHX
P. aeruginosa ATCC 9027	64	32	32	32	64	1,024	64	64	64	4
P. aeruginosa ATCC 27853	32	32	16	32	64	1,024	64	64	64	8
P. aeruginosa M023376	32	64	32	16	64	512	64	64	64	16
P. aeruginosa DM023257	32	64	16	64	64	512	64	32	64	32
P. aeruginosa DM023155	64	32	64	64	64	512	64	64	32	32
S. aureus ATCC 29213	16	32	16	16	1,024	1,024	32	16	2	1
S. aureus ATCC29737	16	128	32	16	1,024	1,024	32	16	2	1
S. aureus DM4001R	32	128	16	16	1,024	1,024	16	16	2	1
S. aureus DM4400R	32	128	32	64	1,024	1,024	32	16	2	1
S. aureus DM4299	16	64	16	16	1,024	512	16	16	4	1
C. albicans ATCC 10231	256	256	128	256	>256	>256	16	16	8	16
C. albicans ATCC 24433	256	256	128	256	>256	>256	16	16	8	8
C. albicans ATCC 2091	256	256	128	256	>256	>256	64	16	4	8
C. albicans DF672R	256	256	128	256	>256	>256	64	16	4	8
C. albicans DF 1976R	256	256	64	256	256	>256	16	16	8	16
Cytotoxicity to hDFs^*b*^	<62.5	<62.5	>2,000	<62.5	<62.5	ND	500	<62.5	2.8	36.5

a Average MIC values from two independent duplicates are reported. ND, not determined.

b Concentration in micrograms per milliliter causing 50% cell death in 24 h (50% inhibitory concentration [IC_50_]), as determined by an MTS assay.

An MTS [3-(4,5-dimethylthiazol-2-yl)-5-(3-carboxymethoxyphenyl)-2-(4-sulfophenyl)-2H-tetrazolium)] assay confirmed the lack of any metabolic activity of hDFs exposed to various concentrations of α-poly-l-lysine (PLL), α-poly-d-lysine (PDL), α-poly-l-ornithine (PLO), PLA, and BPEI, whereas LPEI displayed cytotoxicity at concentrations above 500 μg/ml, and complete viability was observed for ε-polylysine (εPL), even at elevated concentrations (Table 1; see also Fig. S1 in the supplemental material). High-content analysis (HCA) results indicated that hDFs treated with 125 μg/ml of the cationic peptide polymers PLL, PDL, PLO, and PLA displayed deformed cellular morphologies and a complete loss of tubulin fluorescence, which were comparable to those of cells treated with a toxic compound, nocodazole (5 μg/ml), used as a control (Fig. S2a). However, εPL did not show any cytotoxicity, even at elevated concentrations, and hDFs exposed to the εPL polymer did not display any alterations in cellular morphologies or a loss of metabolic activity. Quantitative analysis of multiple morphological parameters that describe the status of cell health, such as cell and nuclear areas, form factor, and cell counts/field, indicated no apparent difference between untreated control cells and cells treated with 1 mg/ml εPL (Fig. S2b). Taken together, the above-described observations suggest that εPL possesses higher microbial cell selectivity over mammalian cells than the other cationic polymers. Among the two ethylenimine polymers, LPEI had higher cell selectivity than BPEI, suggesting that branching could cause a disruption of the cytoskeletal components, possibly by interacting with zwitterionic lipids (22). Between the two cationic antiseptics, BAK displayed heightened toxicity for hDFs compared to CHX. Nevertheless, both antiseptic agents were cytotoxic to hDFs at concentrations close to their MIC values against P. aeruginosa.

### Interaction of cationic polymers with the inner membrane of Gram-negative bacteria.

**FIG 2:**
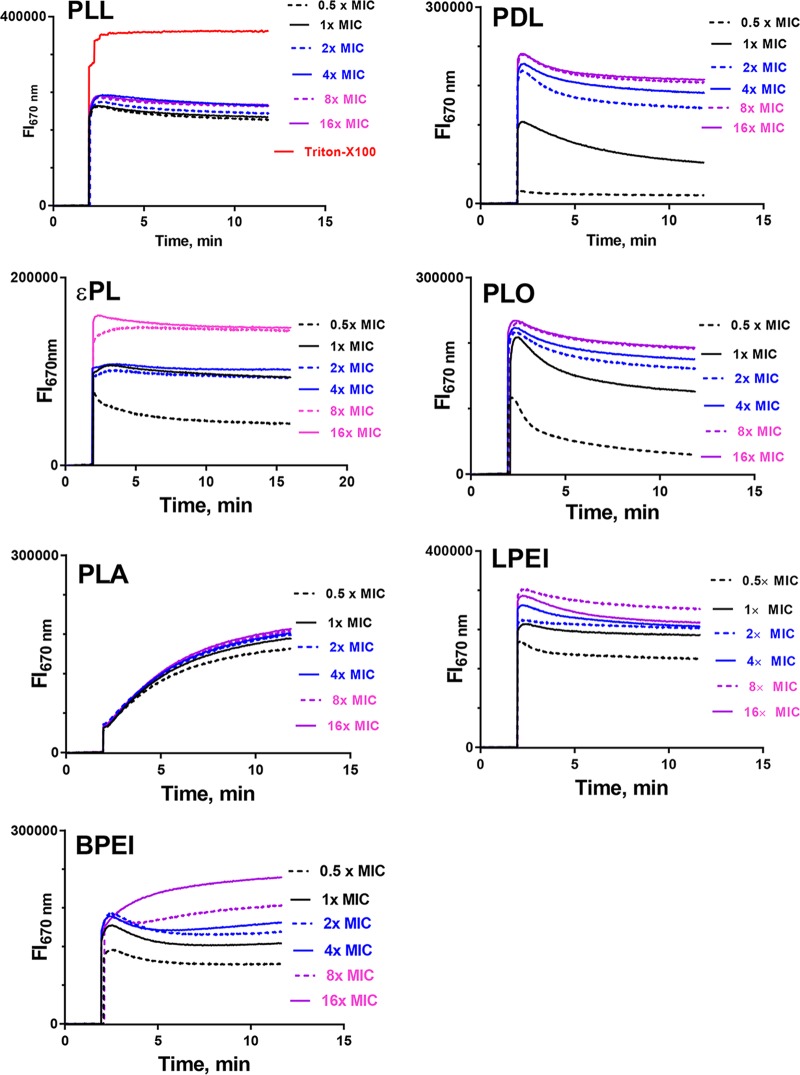
Concentration-dependent changes in the plasma membrane depolarization of P. aeruginosa ATCC 9027 after the addition of cationic polymers. The final concentration of the polymers is expressed in terms of the MIC values. Fl_670 nm_, fluorescence intensity at 670 nm.

To study the mechanism of action, we investigated if the polymers interact with the cytoplasmic membrane of the bacteria and cause a loss of membrane potential, using the potential-sensitive probe DiSC_3_-5 (3,3′dipropylthiadicarbocyanine iodide). Figure 2 indicates the instantaneous loss of membrane potential of P. aeruginosa upon the addition of various cationic polymers. For all the polymers, the loss of membrane potential was rapid, as indicated by the burst release of the potential-sensitive probe from the cytoplasmic membrane. PLL displayed a substantial loss of membrane potential at sub-MICs in comparison to other polymers. εPL, however, showed a weaker perturbation of the cytoplasmic membrane potential at the same concentration. Between the two ethylenimine polymers, LPEI caused a greater release of the potential-sensitive probe than did BPEI. In general, above 2× and 4× MIC, all the polymers caused a substantial loss of membrane potential, suggesting that there was no apparent difference in the bactericidal properties once the MIC values were exceeded. These results indicate that all the cationic polymers perturb the cytoplasmic membrane of bacteria, a mechanism that was akin to that of cationic antimicrobial peptides (23, 24).

### Antibiofilm properties of εPL and LPEI.

**FIG 3:**
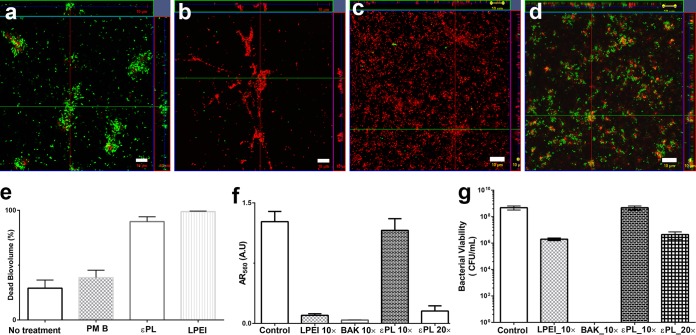
Antibiofilm properties of cationic polymers. A PAO1-gfp biofilm was grown on a microslide for 24 h and treated with various cationic polymers at 10× MIC. The MIC of εPL against this strain was 16 μg/ml. Live/Dead cell staining was imaged by using confocal fluorescence microscopy. (a) Untreated cells; (b) εPL-treated cells; (c) LPEI-treated cells; (d) polymyxin B (PM B)-treated cells; (e) dead biovolume estimated from six different fluorescence images; (f) biomass reduction as shown by a resazurin assay (*AR*_560_) after treatment with the polymers/antiseptic agents for 24 h; (g) bacterial viability after the addition of polymers/antiseptic agents to preformed biofilms. A.U, arbitrary units.

Since εPL and LPEI displayed better selectivity than the other cationic polymers, subsequent experiments were carried out with these two polymers. We investigated the ability of εPL and LPEI to disrupt preformed biofilms of P. aeruginosa strain PAO1 tagged with green fluorescent protein (GFP) (PAO1-gfp). Bacterial biofilm was grown on a flow chamber for 24 h (25). Figure 3a shows confocal images of intact biofilms stained with Live/Dead cell stain. Weakly propidium iodide (PI)-positive cells were observed, indicating that the majority of cells remained intact and alive in the untreated control. However, both cationic polymers at 10× MIC caused a significant increase in PI-positive staining (Fig. 3b to e) and a decrease in the biomass compared to the cationic lipopeptide antibiotic polymyxin B (*P* < 0.0001). Between the two polymers, LPEI displayed a greater reduction in biomass than did εPL (*P* < 0.01). To confirm these antibiofilm properties, resazurin (Fig. 3f) and bacterial viability (Fig. 3g) assays were also carried out using the same strains. The results of both assays, however, indicated that εPL caused a significant (>99%) biomass reduction (>2-log_10_ reduction) only at 20× MIC, and LPEI achieved a similar effect at 10× MIC, whereas BAK achieved the best antibiofilm activity (>8-log_10_ reduction at 10× MIC) in comparison to the other two polymers.

### Bacterial suspension assay.

To ascertain if the two cationic polymers possess antiseptic properties, we determined the reduction factor (*R_f_*) after exposing the polymers to S. aureus, methicillin-resistant S. aureus (MRSA), and P. aeruginosa strains at 10× or 20× MIC for 30 min and 1 h (Table 2). The assay was carried out with Dulbecco's modified Eagle's medium (DMEM) containing 10% fetal bovine serum (FBS) in order to mimic the composition of artificial wound fluid (9). A baseline *R_f_* value of ≥2, which corresponds to a ≥2-log_10_ (99.0%) reduction in the viability of tested organisms in the presence of organic matter, indicates the potency of cationic polymers as antiseptics. For εPL, a longer incubation time was required to achieve baseline *R_f_* values against all the tested organisms than for the cationic antiseptic CHX or BAK. PEIs, however, displayed the strongest activity against MRSA strains only, and higher concentrations or longer incubation times were required to achieve a ≥2-log_10_ reduction of the viability of S. aureus and P. aeruginosa strains. When considering the cytotoxicity of the cationic polymers to mammalian cells, the above-described results suggest that εPL and LPEI possessed a higher biocompatibility index than those of other cationic polymers and cationic antiseptic agents (9).

**TABLE 2:** Biocidal properties of cationic polymers against bacterial strains in the presence of 10% FBS

Polymer	*R_f_*											
	30-min incubation						60-min incubation					
	S. aureus		MRSA		P. aeruginosa		S. aureus		MRSA		P. aeruginosa	
	10× MIC	20× MIC	10× MIC	20× MIC	10× MIC	20× MIC	10× MIC	20× MIC	10× MIC	20× MIC	10× MIC	20**×** MIC
εPL	2.9	3.5	>6	>6	2.0	2.2	3.2	3.3	>6	>6	2.5	2.7
LPEI	2.0	2.1	>6	>6	1.7	1.8	2.7	2.8	>6	>6	2.2	2.3
CHX	3.5	>6	>6	>6	>6	>6	>6	>6	>6	>6	>6	>6
BAK	>6	>6	>6	>6	>6	>6	>6	>6	>6	>6	>6	>6

### Broad-spectrum antimicrobial properties of εPL.

To confirm the broad-spectrum antibacterial properties of εPL, we determined the MIC values of this polymer against a panel of antibiotic-resistant Gram-negative, Gram-positive, and fungal pathogens, which includes bacterial strains that are classified as serious and urgent threats by the Centers for Disease Control and Prevention (26, 27). The results indicated that the MIC values did not shift significantly for various antibiotic-susceptible or antibiotic-resistant bacteria and filamentous fungi (Table 3; see also Table S1 in the supplemental material). The low MIC values against a wide range of Gram-negative, Gram-positive, and filamentous fungal pathogens and the homogenous distribution of MIC values establish the superior broad-spectrum antimicrobial properties of εPL over those of LPEI.

**TABLE 3:** MIC values of εPL against bacteria and fungi^*a*^

Organism(s) (no. of strains)	MIC (μg/ml)
P. aeruginosa (21)	8–32
K. pneumoniae (14)	8–64
A. baumannii (15)	32–64
E. coli (16)	8–32
E. cloacae complex (9)	16–32
S. aureus/MRSA (15)	4–64
Vancomycin-resistant enterococci (21)	4–16
C. albicans (5)	64–128
Fusarium solani and Fusarium oxysporum (7)	<1–64

a MIC values against individual strains are provided in Table S1 in the supplemental material.

**FIG 4:**
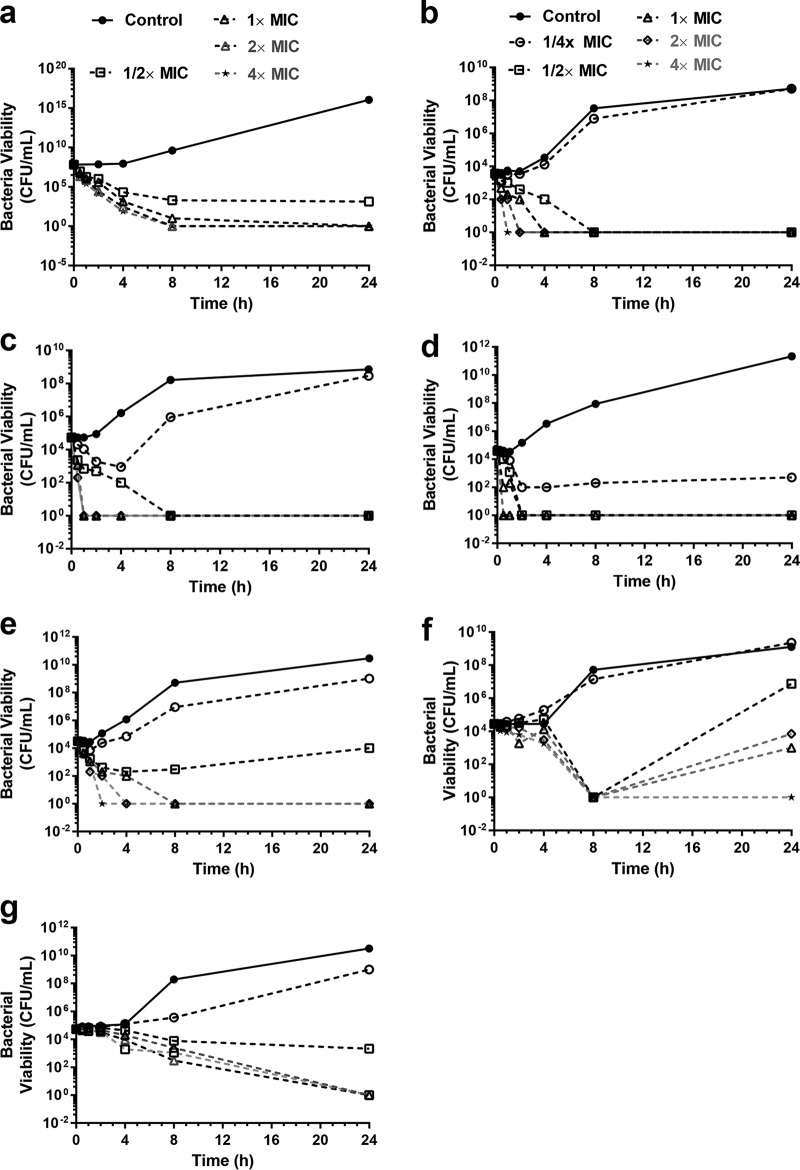
Concentration- and time-dependent bactericidal effect of εPL against various Gram-negative strains. (a) P. aeruginosa ATCC 9027 (16 μg/ml); (b) E. coli ATCC 19211 (16 μg/ml); (c) K. pneumoniae ATCC 55301 (16 μg/ml); (d) A. baumannii 1001 (64 μg/ml); (e) E. cloacae complex strain ATCC 6780 (16 μg/ml); (f) S. aureus 4299 (8 μg/ml); (g) MRSA 9808R (8 μg/ml). MIC values of εPL are shown in parentheses.

Having confirmed the broad-spectrum antibacterial properties, we next investigated the time-kill kinetics of the polymer. εPL displayed concentration-dependent rapid bactericidal activity against antibiotic-resistant Gram-negative strains (Fig. 4a to e). At concentrations above 2× MIC, εPL exposure caused >3-log_10_ reductions in the viability of Escherichia coli, Enterobacter cloacae complex, Klebsiella pneumoniae, and Acinetobacter baumannii strains in 1 h, whereas a slightly longer time was required for P. aeruginosa. However, a much longer time was required to elicit bactericidal activity (>3-log_10_ reduction in viability) against S. aureus and MRSA strains as well (Fig. 4f and g). The rapid bactericidal properties of εPL against antibiotic-resistant Gram-negative strains are advantageous, as they might translate into greater efficacy, reduced emergence of resistance, and reduced duration of treatment.

### Effect of εPL in a rabbit model of corneal wound healing.

**FIG 5:**
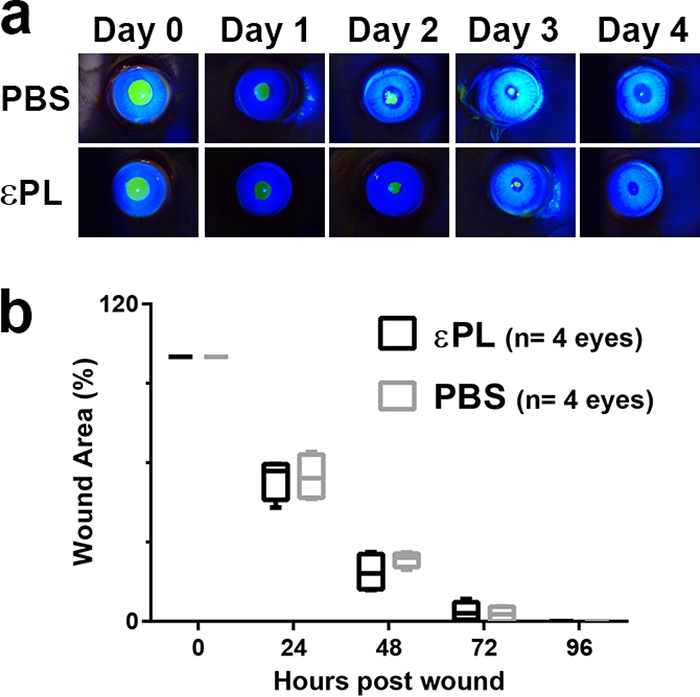
Efficacy of εPL for corneal wound closure *in vivo*. After deepithelialization of the cornea, eyes were treated with PBS (*n* = 4 eyes) or 0.3% (wt/vol) εPL in PBS (*n* = 4 eyes) 4 times/day for 4 days postinjury. (a) Representative slit-lamp images showing the time-dependent changes in wound closure of the cornea after application of εPL or PBS. The wounded cornea was stained with fluorescein to observe epithelial defects. (b) Quantitative estimation of percent wound area after application of εPL or PBS.

We assessed the ocular toxicity of εPL to corneas with epithelial defects in a rabbit model of wound healing (28). After the creation of 6-mm epithelial defects, the injured eyes were treated with 50 μl of 0.3% (wt/vol) εPL in phosphate-buffered saline (PBS) or PBS alone 4 times per day and imaged at 24-h intervals by slit-lamp (SL) microscopy. The results suggested that there was no significant difference between corneas treated with εPL and those treated with the vehicle, confirming that the polymer did not delay the reestablishment of the critical component of corneal innate immunity (Fig. 5).

### Efficacy of εPL in a rabbit model of bacterial keratitis.

**FIG 6:**
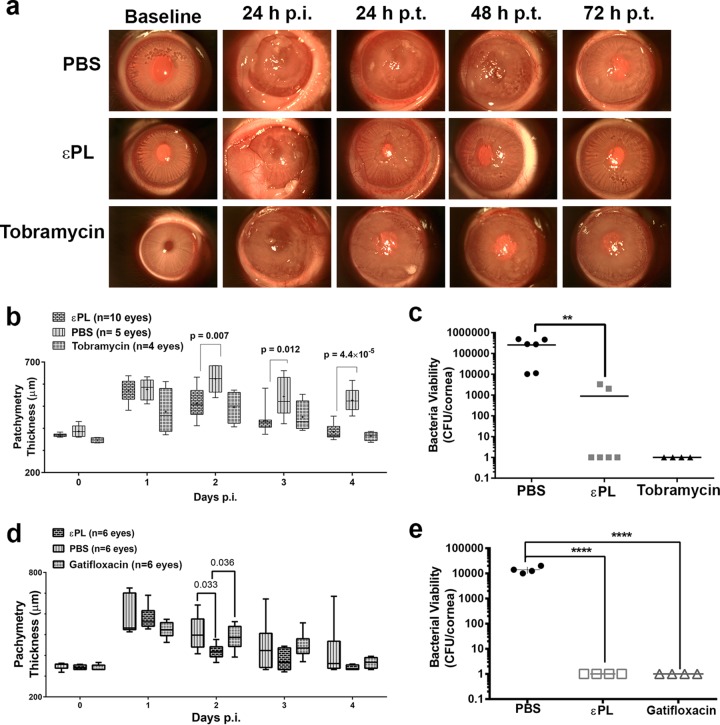
(a) Slit-lamp images showing the efficacy of topical application of εPL in a rabbit model of P. aeruginosa keratitis. Experimental keratitis was induced by P. aeruginosa ATCC 9027. Twenty-four hours after infections, rabbit eyes were treated with 50 μl of PBS, εPL, or tobramycin eye drops (Tobrex) 4 times/day for 3 days. p.i., postinoculation. (b) Time-dependent changes in central corneal thickness (CT) measured by AS-OCT for three different groups after P. aeruginosa infection. Individual CT values for various groups are shown. The horizontal bars represent the mean values. (c) P. aeruginosa burden in infected corneas after treatment of various groups. The results are reported as the means ± standard deviations for 6 corneas per group. (d) Time-dependent changes in central corneal thickness measured by AS-OCT for three different groups after S. aureus infection. The measurement values for different groups are shown. (e) S. aureus burden in infected corneas after treatment of various groups. The results are reported as means ± standard deviations for 6 corneas per group.

We evaluated the efficacy of εPL in a rabbit model of keratitis caused by pathogenic P. aeruginosa and S. aureus strains. Twenty-four hours after infection of the scarified cornea with P. aeruginosa, a 0.3% (wt/vol) εPL solution was applied 4 times per day topically, and the progress of corneal infection before and after the topical application of εPL, PBS, or tobramycin (Tobrex) eye drops was imaged by SL biomicroscopy and anterior-segment optical coherence tomography (AS-OCT). SL photographs indicated that all the eyes (*n* = 10) developed mild to severe infections in the cornea 24 h after inoculation with P. aeruginosa strains (Fig. 6a; see also Fig. S3 in the supplemental material). Significant increases in corneal haze, mucopurulent discharge, and conjunctival chemosis and a steady presence of infiltrates were observed in all infected eyes. However, substantial decreases in chemosis and haze were observed after the topical application of 0.3% εPL at 24 h posttreatment (p.t.). Progressive decreases in conjunctival redness, chemosis, and haze were observed with an increasing treatment duration. AS-OCT was performed to determine the extent of keratitis and the treatment response (Fig. S4). The corneal thickness (CT) increased significantly, from 370.4 ± 7.2 μm to 570.3 ± 49.0 μm, 24 h after inoculation with P. aeruginosa (Fig. 6b), indicating an increased severity of infections. One to three days after treatment with εPL, a progressive decrease in the corneal thickness was observed, confirming the resolution of infection-induced inflammation. The thickness of the cornea reached baseline values after 3 days p.t. for 8/10 corneas treated with εPL, while the other 2 recovered partially (Fig. 6b and Fig. S4). The images furthermore displayed substantial decreases in the number of hyperreflective areas and corneal roughness compared to those of infected corneas treated with PBS, establishing the potency of εPL in clearing P. aeruginosa infections. A similar effect on corneal thickness was observed for infected corneas treated with tobramycin eye drops. The average corneal thickness was significantly decreased in the εPL-treated groups (*P* = 0.007) by as early as 24 h p.t. compared to those of the PBS-treated groups, although no statistical difference (*P* ≤ 0.05 is considered statistically significant) was observed between εPL- and tobramycin-treated corneas (*P* = 0.66 at 1 day p.t., *P* = 0.65 at 2 days p.t., and *P* = 0.36 at 3 days p.t.).

Finally, microbiological determination of bacterial viability indicated that there were no viable bacteria in 5/6 corneas after εPL treatment, whereas in one cornea, 3.4 ± 0.12 log_10_ CFU were observed (Fig. 6c). However, PBS-treated corneas contained 5.1 ± 0.7 log_10_ CFU, suggesting a substantial decrease in the bacterial burden after εPL treatment. Together with data from *in vitro* microbiological studies, these results establish the excellent antimicrobial properties of εPL for P. aeruginosa keratitis.

An even more dramatic antimicrobial effect was observed for S. aureus-infected corneas upon the topical application of εPL. SL examination and AS-OCT images of eyes treated with εPL indicated marked decreases in chemosis, discharge, corneal haze, conjunctival redness, as well as corneal edema in comparison with the other groups (Fig. S5 and S6). A complete recovery of the baseline corneal thickness was achieved at 72 h p.t. with εPL. AS-OCT images confirmed that εPL-treated eyes displayed significant decreases in thickness at 24 h p.t. (418.2 ± 14.5 μm) in comparison to gatifloxacin (Zymar)-treated eyes (486.8 ± 24.2 μm) (*P* = 0.036) or PBS-treated eyes (508.5 ± 33.5 μm) (*P* = 0.033) (Fig. 6d). Microbiological studies confirmed that both εPL-and gatifloxacin-treated corneas did not show the presence of viable bacteria, whereas PBS-treated corneas contained 4.1 ± 0.13 log_10_ CFU/cornea (Fig. 6e). These results demonstrate that the topical application of εPL reduced clinical symptoms as well as bacterial bioburdens in both P. aeruginosa and S. aureus models of infectious keratitis.

## DISCUSSION

The rapid upsurge in the evolution of antibiotic-resistant bacteria undermines the antibiotic armamentarium and endangers the benefits achieved with antibiotics. Recent studies showed that cationic antimicrobial polymers are potent alternatives for combating drug-resistant pathogens, owing to their rapid bactericidal and membrane-targeting actions. Several synthetic and natural polymers display potent antimicrobial activity against both antibiotic-susceptible and antibiotic-resistant pathogens, but their efficacy as biocides for topical applications remains uncertain. In this study, we compared the antimicrobial activities and biocompatibilities of polymers with three different backbone architectures. The cationic polymers displayed moderate to weak antimicrobial properties (in terms of MIC values) against the tested P. aeruginosa, S. aureus, and C. albicans strains in comparison to cationic antiseptics. The results suggested that the antimicrobial properties are governed by both backbone (PEIs versus PAA) and side-chain (polylysines/PLO/εPL versus PLA) groups. Although the antimicrobial properties of the polymers appeared weaker than those of antiseptics, estimation of the MIC values in terms of molar concentrations suggests that the polymers are as potent as the topical antiseptics. MTS assays and HCAs revealed that εPL and LPEI displayed superior cell selectivity over other cationic polymers. εPL and LPEI furthermore displayed potent antibiofilm activities against preformed P. aeruginosa biofilms at a concentration that was less toxic or nontoxic to hDFs compared to BAK, demonstrating their efficacy in reducing the bacterial biovolume. Both polymers caused a substantial loss of membrane potential, indicating that the antimicrobial actions of polymers involve the disruption of the cytoplasmic membrane. Bacterial suspension assays confirmed that both polymers retained their antimicrobial properties in the presence of FBS, and their biocompatibility indexes were superior to those of CHX and BAK (9). Taken together, these results imply that the two polymers can be useful as biocides for treating infections caused by pathogenic bacteria.

Interestingly, LPEI displayed potent antimicrobial activities against P. aeruginosa and S. aureus/MRSA strains only: the MIC values were >128 to 256 μg/ml when it was tested against other Gram-negative strains such as A. baumannii, K. pneumoniae, and E. coli as well as against Gram-positive strains such as Bacillus subtilis and Enterococcus faecalis (22). The origins of the P. aeruginosa- and S. aureus-specific activity of LPEI are not clear and require detailed scrutiny. It is tempting, however, to suggest that the changes in zeta potentials of different Gram-negative bacteria could be responsible for the observed higher MIC values for LPEI (29, 30). Among the various polymers, the biosynthetic polymer εPL displayed a homogeneous distribution of MIC values against several antibiotic-resistant pathogens that have been listed as serious threats to human health by the CDC. Data from time-kill kinetics assays suggested that εPL caused substantial losses of viability of various Gram-negative bacteria in less than 2 h and at values above 2× MIC, suggesting that membrane depolarization was lethal to the bacteria. This polymer also displayed potent antifungal activities against the ocular fungal pathogen Fusarium, and the values were comparable to those for the ophthalmic antifungal natamycin. Together with the data from the cell suspension assay, these results further establish the broad-spectrum biocidal properties of εPL.

The *in vivo* toxicity and efficacy of εPL were established in rabbit models of corneal wound healing and infectious keratitis, respectively. The topical application of εPL did not affect the reepithelialization rate in an injured cornea, confirming the ocular safety of this polymer. Efficacy studies revealed that εPL not only decreased the bacterial burden but also decreased the severity of infection substantially, as confirmed by AS-OCT results. Antimicrobial efficacy was more pronounced for S. aureus keratitis than for corneas infected with P. aeruginosa, as indicated by the substantial decrease in corneal edema at 24 h p.t. with εPL. SL examination, AS-OCT, and microbiological studies confirmed that there was no apparent difference in the antimicrobial activities between topical antibiotic eye drop formulations and εPL. Previous studies have shown that povidone-iodine or CHX topical antiseptic formulations had weaker *in vivo* efficacy and led to poorer clinical outcomes than antibiotic eye drops (31–35). Based on this, we suggest that εPL can be used as an alternative therapy for the treatment of superficial bacterial infections as well as antimicrobial prophylaxis. Its poor systemic absorption, excellent safety profile for mammalian cells, noninterference with the reepithelialization of injured corneas, broad-spectrum antimicrobial properties, *in vivo* efficacy in eradicating infections and the concomitant severity, and cost-effectiveness are additional advantages.

In summary, our studies establish the broad-spectrum antimicrobial properties, rapid bactericidal and antibiofilm properties, high biocompatibility index, and *in vivo* efficacy of the biosynthetic polymer εPL. This polymer is classified as “generally regarded as safe” (GRAS) by the U.S. FDA and is used as a food preservative in the United States, South Korea, and Japan. Our results demonstrate that εPL is a promising antiseptic/antimicrobial agent for treating ocular as well as superficial bacterial infections.

## MATERIALS AND METHODS

### Materials.

εPL (*M_w_*, ∼4,000) was purchased from Hefei TNJ Chemical Industry Co Ltd., China. PLL (*M_w_*, 15,000 to 30,000), PLO (*M_w_*, 15,000 to 30,000), PDL (*M_w_*, 30,000 to 70,000), PLA (*M_w_*, >70,000), PAA (*M_w_*, ∼65,000), LPEI (*M*_n_, ∼20,000), BPEI (*M*_n_, ∼10,000), and BAK (360 g/mol) were purchased from Sigma-Aldrich Pte. Ltd., Singapore. All the chemicals were of analytical grade and used without any further purification. Rinscap CG, which contains CHX gluconate (897.8 g/mol; 0.05%, vol/vol), was obtained from Joyson Pte. Ltd. We used this solution as it is for our studies. All the polymers or cationic antiseptics were dissolved readily in aqueous media, and a 10-mg/ml stock solution in water was used for microbiology and cytotoxicity studies.

### MIC determination.

MICs of cationic polymers were tested against a panel of antibiotic-susceptible/antibiotic-resistant bacterial, yeast, and fungal strains in accordance with Clinical and Laboratory Standards Institute (CLSI) guidelines (42) (see Table S1 in the supplemental material). Bacterial and yeast strains were cultured on tryptic soy agar (TSA) and Sabouraud dextrose agar (SDA) plates (Neogen Corporation, MI, USA) overnight, respectively. Inocula in Mueller-Hinton broth (MHB) for bacteria and Sabouraud dextrose (SD) broth for C. albicans (Becton Dickinson, MD, USA) were prepared at a 0.5 McFarland standard. The suspensions were then diluted to a final concentration of 10^5^ CFU/ml in a 96-well microtiter plate (SPL Life Sciences Co., Ltd., South Korea). Polymers were added to the inoculum in 2-fold serial dilutions to give a range of concentrations from 2 to 1,024 μg/ml. MICs of the polymers were determined after 24 h of incubation at 35°C by measuring the optical density at 600 nm (OD_600_) using a Tecan Infinite M200 microplate reader (Tecan, Austria) as well as by visual observation. Antimicrobial activities were compared with those of the topical antiseptic agents BAK and CHX. A similar protocol was used to determine the MIC of εPL against antibiotic-resistant pathogens. The MIC of the polymer against Fusarium strains was determined with full-strength RPMI 1640 buffer. Fungal spores were recovered from a 5-day-old culture on potato dextrose agar and diluted to a concentration of 10^5^ spores/ml in a 0.9% saline solution. A further 50-fold dilution was done in RPMI 1640 buffer, and 100 μl of the inoculum was added to 96-well plates containing an equal volume of test peptides at 2-fold serial dilutions. Two hundred microliters of the inoculum without any additives and buffer alone served as positive and negative controls, respectively. The plate was then incubated at 30°C for 48 h. The absorbance value was measured at 600 nm as described above, and the lowest concentration of the peptide which inhibited 90% growth was reported. All MIC determinations were performed in duplicates.

### Time-kill kinetics.

The kinetics of the bactericidal action of εPL was determined against 5 Gram-negative and 4 Gram-positive strains. The time-kill studies were performed with a final inoculum of 10^4^ to 10^6^ CFU/ml in MHB with the peptides/antibiotics at 1/2×, 1×, 2×, and 4× the MIC values. The tubes were incubated at 37°C under continuous agitation. Duplicate samples were withdrawn at various time intervals (0 to 24 h), and the log_10_-fold dilutions were plated onto a TSA plate for CFU enumeration.

### Cytoplasmic membrane depolarization (DiSC_3_-5) assay.

Suspensions of P. aeruginosa ATCC 9027 cells grown overnight were harvested, resuspended in 5 mM HEPES buffer at pH 7.4, and adjusted to an OD_600_ of 0.2. The cytoplasmic membrane potential-sensitive probe DiSC_3_-5 (Sigma-Aldrich, MO, USA) was added to the inoculum to a final concentration of 10 μM, and the mixture was incubated at room temperature for 1 h. The suspension was transferred into a 10-mm stirring quartz cuvette, and 1 μl of each polymer in different concentrations was added after a stable signal was detected. The fluorescence intensity at excitation and emission wavelengths of 622 nm and 670 nm, respectively, was monitored until a plateau was reached by using a Quanta Master spectrophotometer (Photon Technology International, NJ, USA) with a slit width of 0.5 nm. The fluorescence intensity values after the addition of Triton X (0.1%, wt/vol) was taken as maximum depolarization, and the percentage of depolarization was calculated.

### Bacterial suspension assay.

To determine the antiseptic properties of the polymers, we followed the protocol reported previously by Müller and Kramer (9). S. aureus strain ATCC 29213, MRSA strain ATCC 700699, and P. aeruginosa strain ATCC 9027 were used in this study. Briefly, a bacterial suspension was prepared in MHB with a final inoculum of 10^8^ to 10^9^ CFU/ml. One hundred microliters of this suspension was transferred to a 900-μl mixture of DMEM (containing 10% FBS) with cationic polymers at a final concentration of 10× or 20× MIC values. A bacterial inoculum added to DMEM containing antiseptics (BAK or CHX) and DMEM (containing 10% FBS) alone served as positive and growth controls, respectively. The whole mixture was incubated for 30 min or 1 h at 35°C. The microbicidal activity of the mixture was inactivated by adding 100 μl of the mixture to 900 μl of TLA-thio (made of 3% [wt/vol] Tween 80 [Sigma-Aldrich], 0.3% [wt/vol] lecithin from soy bean, 0.1% [wt/vol] histidine [Sigma-Aldrich], and 0.5% [wt/vol] sodium thiosulfate [Sigma-Aldrich]), and the mixture was incubated for 30 min at room temperature. Tenfold serial dilutions were prepared in Trypticase soy broth (Neogen Corporation). One hundred microliters of each dilution was plated in duplicate onto Trypticase soy agar plates. The plates were incubated for 48 h at 35°C, and bacterial colonies were enumerated. The reduction factor was evaluated by using the formula *R_f_* = log_10_ *N_c_* − log_10_ *N_d_*, where *N_c_* and *N_d_* are the numbers of viable cells remaining after exposure of the inoculum to the medium alone and to the medium containing the cationic polymers and antiseptics, respectively.

### Antibiofilm activity of cationic polymers.

P. aeruginosa PAO1-gfp bacteria (which constitutively express green fluorescent protein) were incubated in a shaking incubator at 37°C. Cultures grown overnight were adjusted to an OD_600_ of 0.1 prior to inoculation into μ-Slide 8-well glass-bottom plates (ibidi, Germany). Biofilms were grown in three different 8-well chambers at 37°C in ABT minimal medium supplemented with 0.4 g of glucose per liter of medium (ABTG medium) for 24 h. At the end of day 1, the chamber wells were gently washed with sterile PBS twice and replaced with fresh ABTG medium containing the respective antimicrobial polymers (10× to 40× MIC), while the control chamber was replaced with just fresh ABTG medium. After 24 h, in one of the 8-well chambers, ABTG medium containing 20 μM PI from a Live/Dead BacLight kit (Molecular Probes Inc., OR, USA) was supplied to stain the dead biofilms. Confocal images were taken by using a Zeiss LSM780 confocal laser scanning microscope (Carl Zeiss, Jena, Germany) at excitation and emission wavelengths of 488 nm and 535 nm for GFP and 561 nm and 617 nm for PI stain, respectively. Confocal images were analyzed by using IMARIS software to obtain live/dead cell ratios in the biofilm. In the second 8-well chamber, 200 μl of ABTG medium containing 0.1 mM resazurin was added to each well, and the mixture was incubated for 2 h at 37°C (36). One hundred microliters of this solution was transferred to a fresh 96-well plate, the amount of reduced resazurin (resorufin) was determined by monitoring the absorbance at 560 nm, residual amounts of oxidized resazurin were quantified by measuring the absorbance at 620 nm using a Tecan Infinite M200 microplate reader (Tecan, Austria), and the corrected *A*_560_ (*AR*_560_) values were calculated by using the following formulas: *AR*_560_ = *A*_560_ − (A_620_ × *R*_0_), where *R*_0_ = *AO*_560_/*AO*_620_; *AO*_560_ and *AO*_620_ − absorbance of ABTG medium + 0.1 mM resazurin at 560 and 620, respectively; and *A*_560_ and *A*_620_ − absorbance of the polymer samples. The lower the *AR*_560_ value, the greater the inhibition of bacterial growth or biofilm disruption.

In the third 8-well chamber, 200 μl PBS was added to the wells, and the biofilm was removed by gentle scraping followed by pipette mixing and transferred to 1.5-ml Eppendorf tubes. This solution was vortexed with glass beads to completely disrupt the biofilm, and 100 μl of this suspension was serially diluted and plated. The plates were incubated at 35°C for 24 h before enumeration of the colonies. The average values from two independent duplicate experiments are reported.

### Cytocompatibility assessment of polymers.

An MTS assay and an HCA were performed to elucidate the effects of various polymers on the metabolic activity and morphological parameters of cultured hDFs, respectively, as described previously (22). The antineoplastic agent nocodazole (5 μg/ml) served as the negative control, whereas cells treated with PBS served as the positive control. The average values from three independent triplicate experiments are reported.

Cells were cultured in 96-well plates and scanned (16 randomly selected fields/well) by using an IN Cell Analyzer 2200 automated microscope (GE Healthcare). The multiparametric cytotoxicity bioapplication module of the IN Cell Investigator software (GE Healthcare) was used for quantitative estimations and morphotypic analysis of acquired images, which were automatically converted into color-coded heat maps by using Spotrfire software (22, 37, 38).

### *In vivo* biocompatibility of εPL in a rabbit model of corneal epithelial wound healing.

All the animals used for the study of the *in vivo* biocompatibility of εPL were treated in accordance with the tenets of the Association for Research in Vision and Ophthalmology (ARVO) statement (43), and the protocol was approved by the SingHealth Institutional Animal Care and Use Committee (IACUC) (AALAC accredited; protocol number 2012/SHS/775 for wound healing and protocol number 2014/SHS/1010 for bacterial keratitis studies). Eight New Zealand White rabbits, aged 5 months (body weight, 3 to 3.5 kg), were used for this study and divided into two groups. Prior to wounding, all rabbit eyes were examined by slit-lamp photography for the absence of corneal aberrations such as vascularization or any other ocular surface defects. A 6-mm-diameter circular region of the corneal surface was deepithelialized with a sterile miniblade (Beaver; BD, MA, USA) after the rabbits were anesthetized. The two groups of rabbits received a 50-μl topical instillation of 0.3% (wt/vol) εPL (in PBS, pH 7.0) or PBS 4 times a day until complete wound closure was observed. Corneal epithelial wound healing was visualized by the addition of a drop of 2% (wt/vol) sodium fluorescein (Bausch & Lomb), which revealed epithelial defects upon illumination with a cobalt blue filter, and photographed immediately after wounding as well as 1, 2, 3, and 4 days after injury. The area of the epithelial defects was then estimated by using ImageJ software.

### *In vivo* efficacy of εPL in P. aeruginosa and S. aureus models of infectious keratitis.

New Zealand White rabbits weighing 2 to 2.5 kg were used for the study of the *in vivo* efficacy of εPL. The rabbits were anesthetized, and the corneal surface was deepithelialized with a sterile miniblade (Beaver; BD, MA, USA). Corneal infection was induced by applying 50 μl of 5 × 10^6^ CFU/ml S. aureus strain ATCC 29213 or P. aeruginosa strain ATCC 9027 to the scarified cornea. At 24 h postinfection, 50 μl of 0.3% (wt/vol) εPL (in PBS, pH 7.0) or PBS was applied topically to the infected eyes 4 times/day. Tobramycin eye drops (Alcon, Belgium), which contain 0.3% tobramycin, served as the positive control for P. aeruginosa keratitis, whereas gatifloxacin (Allergan, USA) eye drops were used as a positive control for S. aureus keratitis. Slit-lamp photographs and AS-OCT scans were taken before and after infection as well as during the course of treatment (39, 40). The preinoculation and postinoculation corneal thicknesses (CTs) were measured perpendicular to the anterior corneal surfaces, and the average CT was reported (41).

### Quantification of viable bacteria.

Three days after treatment with εPL, ophthalmic antibiotic eye drops, or PBS, rabbit corneas were removed by trephination and homogenized individually in sterile PBS by using plastic pestles, followed by finer homogenization with bead beating by using sterile 2-mm-diameter glass beads. Bacterial enumeration was carried out by spreading the homogenate (10- and 100-fold serial dilutions) on TSA plates, and the plates were incubated for 48 h at 37°C.

## Supplementary Material

Supplemental material
