# Supplementary material for: Antimicrobial Activity and Cell Selectivity of Synthetic and Biosynthetic Cationic Polymers
Source: Antimicrob Agents Chemother. 2017 Sep 22;61(10):e00469-17. doi: 10.1128/AAC.00469-17 (PMC5610535; doi:10.1128/AAC.00469-17)
Supplement: Supplemental material [file supp_61_10_e00469-17__index.html]

Supplemental material 

# Antimicrobial Activity and Cell Selectivity of Synthetic and Biosynthetic Cationic Polymers

## Supplemental material

- Supplemental file 1 -

  Supplemental Figures S1 to S6

  PDF, 1.8M
- Supplemental file 2 -

  Supplemental Table S1

  XLSX, 26K
