# Supplementary material for: Antimicrobial Activity and Cell Selectivity of Synthetic and Biosynthetic Cationic Polymers
Source: Antimicrob Agents Chemother. 2017 Sep 22;61(10):e00469-17. doi: 10.1128/AAC.00469-17 (PMC5610535; doi:10.1128/AAC.00469-17)
Supplement: Supplemental material [file AAC.00469-17_zac010176592s1.pdf]

Antimicrobial Activity and Cell Selectivity of Synthetic and Biosynthetic Cationic Polymers

Mayandi Venkatesh,[a†] Veluchamy Amutha Barathi,[a,b†] Eunice Tze Leng Goh,[a] Raditya Anggara,[a] Mobashar Hussain Urf Turabe Fazil,[c] Alice Jie Ying Ng,[a] Sriram Harini,[a] Thet Tun Aung,[a] Stephen John Fox,[d] Shouping Liu,[a,b] Liang Yang,[e,f] Timothy Mark Sebastian Barkham,[g] Xian Jun Loh,[h] Navin Kumar Verma,[c#] Roger W Beuerman,[a,b#] Rajamani Lakshminarayanan[a,b#]

Anti-Infectives Research Group, Singapore Eye Research Institute, The Academia, 20 College Road, Discovery Tower, Singapore[a]; Ophthalmology and Visual Sciences Academic Clinical Program, Duke-NUS Graduate Medical School, Singapore[b]; Lee Kong Chian School of Medicine, Nanyang Technological University, Experimental Medicine Building, Singapore[c]; Bioinformatics Institute, Agency for Science, Technology and Research (A*STAR), Singapore[d]; Singapore Centre for Environmental Life Sciences, Nanyang Technological University, Singapore[e]; School of Biological Sciences, Nanyang Technological University, Singapore[f]; Department of Laboratory Medicine, Tan Tock Seng Hospital, 11 Jalan Tan Tock Seng, Singapore[g]; Institute of Materials Research and Engineering, A*STAR (Agency for Science, Technology and Research), 2 Fusionopolis Way. Innovis 08-03, Singapore[h].

Running Head: Membrane Selectivity of Cationic Polymers

[†]These authors contributed equally to this work

#Address correspondence to Dr. Rajamani Lakshminarayanan (lakshminarayanan.rajamani@seri.com.sg), Roger W Beuerman (roger.wilmer.beuerman@seri.com.sg) and Navin Kumar Verma (nkverma@ntu.edu.sg)

Fig. S1

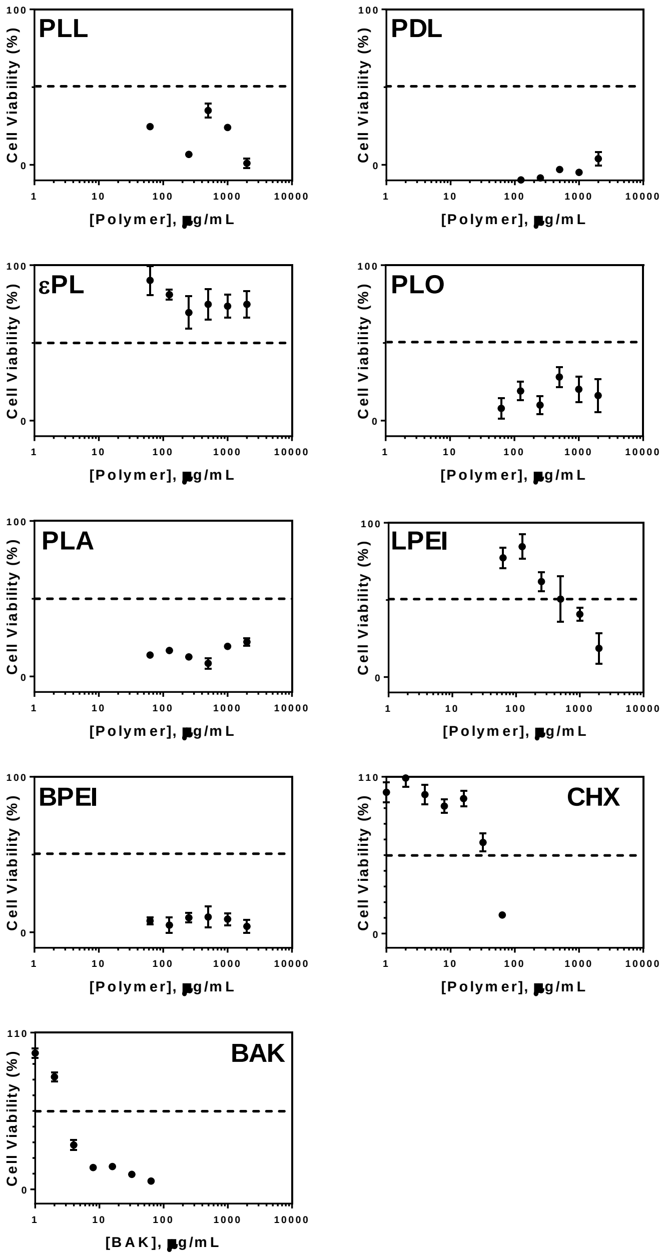

Fig. S1. Cytotoxicity of cationic polymers for hDFs determined by MTS assay. For a comparison, cationic antiseptics chlorohexidine (CHX) and benzalkonium chloride (BAK) were also included. The cells were exposed to various concentrations of the polymers for 24 h and their metabolic activity was quantified by colorimetric changes in MTS. The colorimetric read out was converted to % cell viability from two independent triplicate experiments. The horizontal dotted lines indicate 50% viability.

Fig. S2

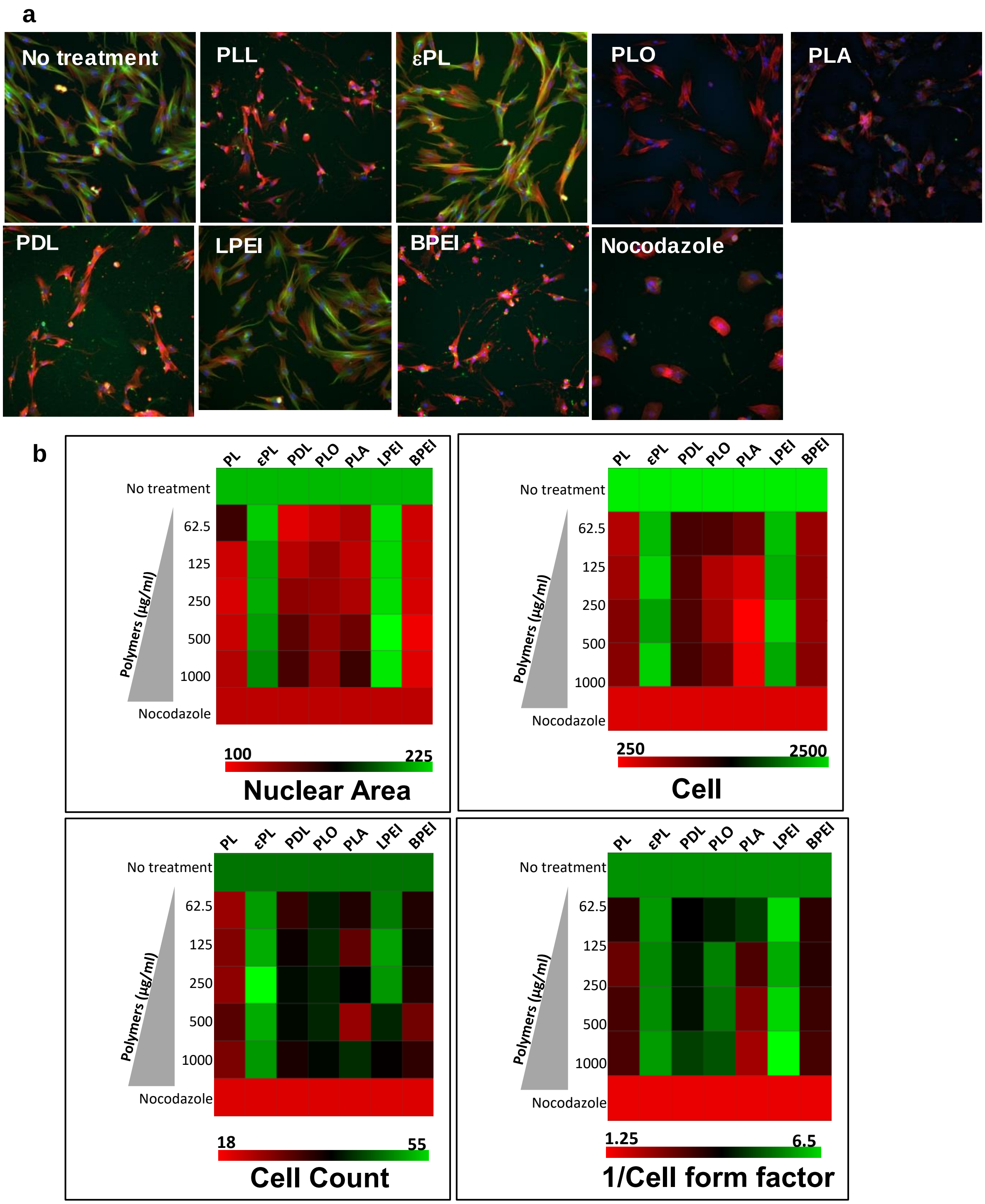

Fig. S2. **a**) Immunofluorescence images showing the morphology of primary human dermal fibroblasts after exposure to the cationic polymers for 24 h. Cells were treated with 125 μg/ml of PLL, PDL, PLO, PLA and BPEI. The images display the effect of LPEI and εPL at a concentration of 500 μg/ml. Cells were

stained FITC-conjugated anti-α-tubulin (green) and rhodamine-phalloidin for actin (red) and Hoechst (blue). **b**) Heat maps showing the concentration-dependent changes in the morphological parameters of hDFs after exposure to various cationic polymers for 24 h. Nocodazole (5 μg/ml) served as the negative control while untreated cells served as positive controls. Note the lack of changes in the parameters for εPL and LPEI, suggesting their good biocompatibility.

Fig. S3

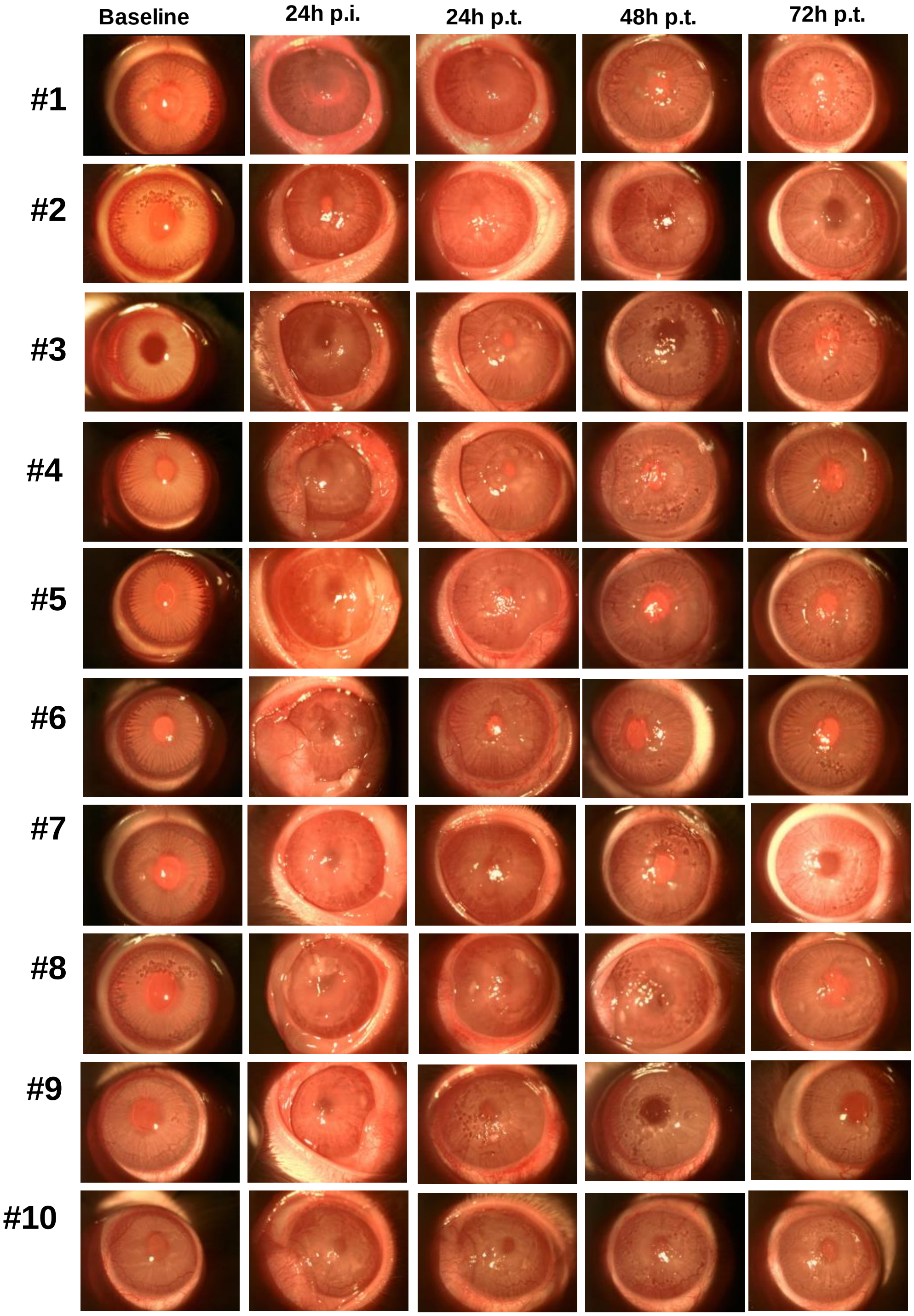

Fig. S3 Slit lamp photographs showing the efficacy of 0.3% (w/v) εPL in a rabbit model of *Pseudomonas* keratitis. Note that a dramatic decrease in conjunctival edema, redness and corneal haze after topical instillation of the polymer.

Fig. S4

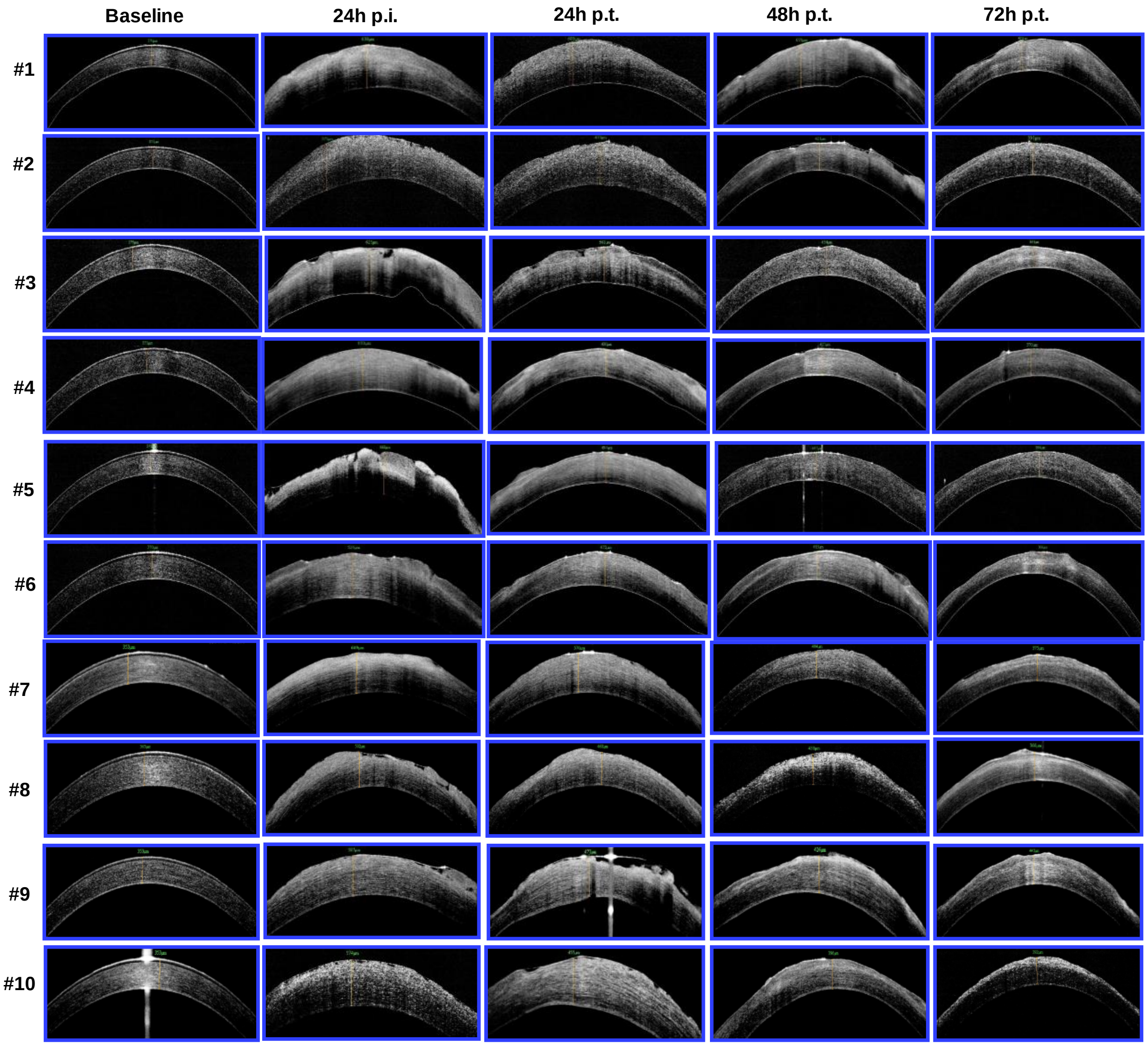

Fig. S4 AS-OCT images showing the transverse efficacy of 0.3% (w/v) εPL in a rabbit model of *Pseudomonas* keratitis. Note that a dramatic decrease in corneal edema and hyper-reflective substances after the addition of εPL.

Fig. S5

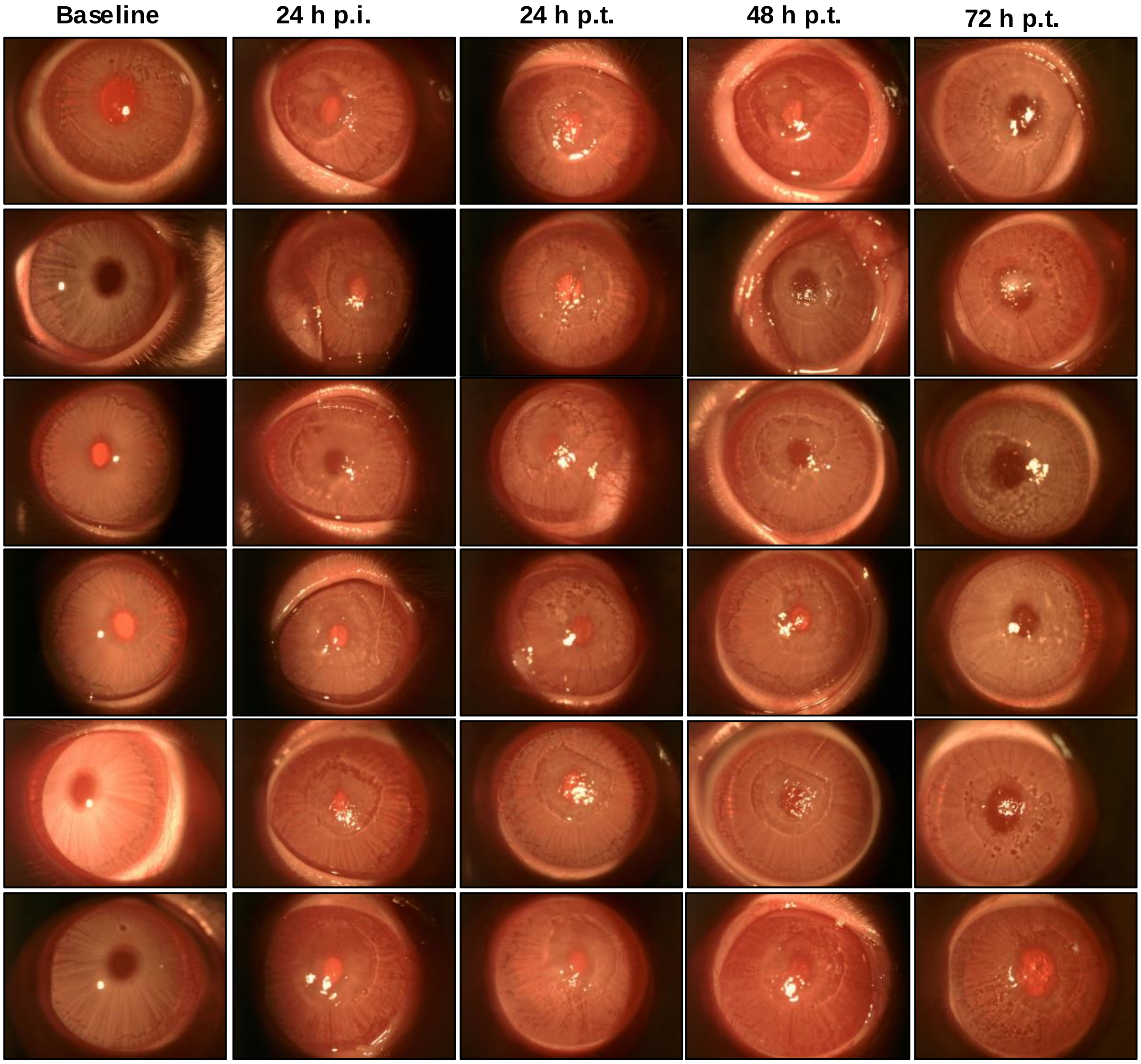

Fig. S5. Antimicrobial efficacy of εPL in a *S. aureus* keratitis. Slit lamp photographs showing the progressive changes in the cornea before and after topical instillation of 0.3% εPL (w/v).

Fig. S6

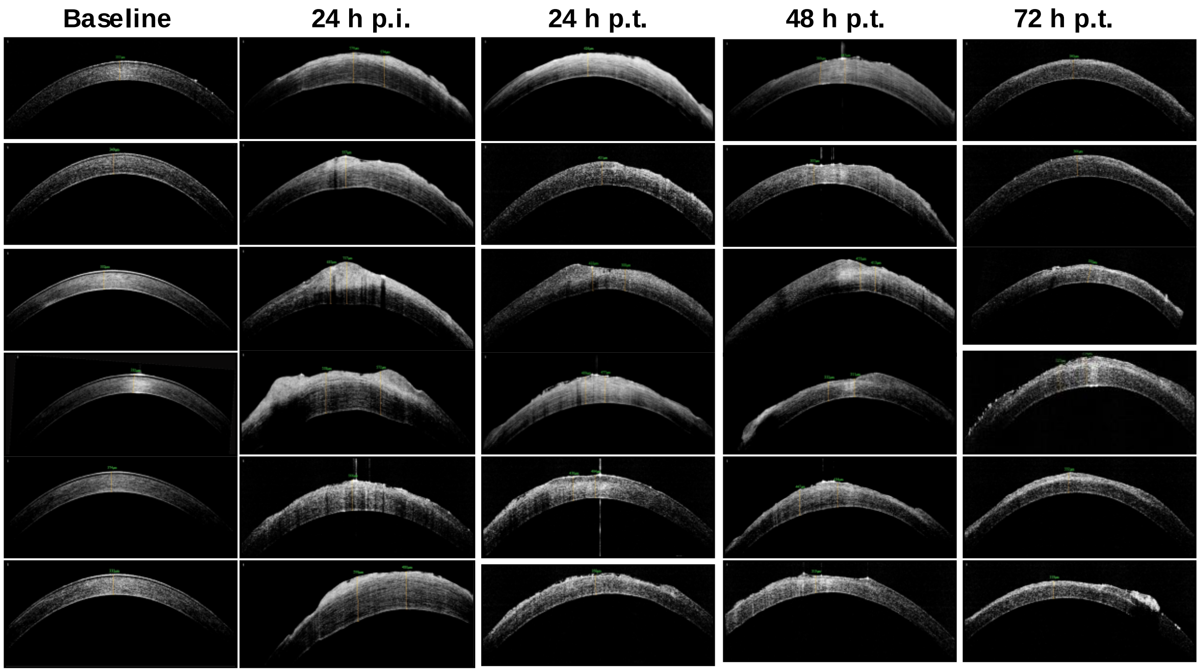

Fig. S6 Antimicrobial efficacy of εPL in a *S. aureus* keratitis. AS-OCT images showing the progressive changes in the corneal thickness before and after infections as well as after treatment with 0.3% εPL (w/v).
